# Severe Disease Caused by Community-Associated MRSA ST398 Type V, Australia, 2017

**DOI:** 10.3201/eid2501.181136

**Published:** 2019-01

**Authors:** Geoffrey W. Coombs, Stanley Pang, Denise A. Daley, Yung Thin Lee, Sam Abraham, Marcel Leroi

**Affiliations:** PathWest Laboratory Medicine WA, Murdoch, Western Australia, Australia (G.W. Coombs);; Murdoch University, Murdoch (G.W. Coombs, S. Pang, Y.T. Lee, S. Abraham);; Australian Group on Antimicrobial Resistance, Murdoch (D.A. Daley);; Austin Health, Heidelberg, Victoria, Australia (M. Leroi)

**Keywords:** methicillin-resistant *Staphylococcus aureus*, *Staphylococcus aureus*, endocarditis, sepsis, livestock, MRSA, Australia, ST398, severe disease, bacteria, antibiotic resistance, MRSA and other staphylococci, livestock-associated MRSA, Australian *Staphylococcus* Sepsis Outcome Programme, community-associated MRSA, SCC*mec* V, V (5C2&5), PVL negative, t011, *spa* type, human evasion genes

## Abstract

Using whole-genome sequencing, we identified a community-associated methicillin-resistant *Staphylococcus aureus* (CA-MRSA) sequence type (ST) 398 type V (5C2&5) isolate (typically found in China) in Australia in 2017. This CA-MRSA ST398 variant was highly virulent, similar to other related CA-MRSAs of ST398. This strain should be monitored to prevent more widespread dissemination.

The Australian Group on Antimicrobial Resistance (http://agargroup.org.au/) manages multiple national antimicrobial drug resistance surveillance programs, including the Australian *Staphylococcus* Sepsis Outcome Programme. This program involves 38 hospitals across Australia continuously providing antimicrobial MIC data and demographic data on episodes of *Staphylococcus aureus* sepsis. Specimens are referred to a central reference laboratory where whole-genome sequencing is performed for all methicillin-resistant *S. aureus* (MRSA) isolates.

In 2017, a MRSA sequence type (ST) 398 type V (5C2&5) isolate, typically referred to as livestock-associated MRSA (LA-MRSA), which has been isolated in many parts of the world including Australia (*1*,*2*), was cultured from a 56-year-old man from Singapore who was working as a chef in a suburb of Melbourne, Victoria, Australia. He sought hospital care for a 2-week history of a nonspecific illness and was found to have mitral valve endocarditis with embolic disease involving the spleen, brain, and lungs. Because his MRSA bacteremia failed to resolve after 10 days of vancomycin therapy, he required a mechanical mitral valve replacement. His bacteremia resolved 24 hours after valve replacement. Because of glycopeptide hypersensitivity and concerns of vancomycin heteroresistance, he was prescribed various nonglycopeptide antimicrobial drug therapies (e.g., clindamycin monotherapy, moxifloxacin monotherapy) for variable durations throughout the remainder of his treatment. While he was in the hospital, acute renal injury developed, requiring hemodialysis support. After 3 months’ hospitalization, he was transferred to a hospital in Singapore. At the time of transfer, he was improving and undergoing rehabilitation. We were not able to establish if the patient had direct contact with livestock, but as a chef, he presumably had contact with raw meat.

We identified the patient’s isolate (S23009-2017) as *S. aureus* by matrix-assisted laser desorption/ionization time-of-flight mass spectrometry using the Bruker MALDI Biotyper (https://www.bruker.com/) and confirmed this finding by DNA microarray using the *S. aureus* Genotyping Kit 2.0 (Alere Technologies, https://alere-technologies.com/). We performed susceptibility testing by using the VITEK 2 automated microbiology system (bioMérieux, https://www.biomerieux.com/) and performed whole-genome sequencing of the isolate using the Illumina NextSeq 550 Sequencing System (https://www.illumina.com/). We performed DNA extraction on an overnight subculture using the Invitrogen MagMAX DNA Multi-Sample Kit (ThermoFisher, https://www.thermofisher.com/). We prepared a library of the extracted DNA using the Illumina Nextera XT Library Preparation Kit and sequenced libraries having 150-bp paired-end chemistries. We identified single-nucleotide polymorphisms and performed core genome alignments using Snippy version 3.2 (https://github.com/tseemann/snippy). We constructed a phylogenetic tree using the resulting single-nucleotide polymorphisms in MEGA version 7.0 (https://www.megasoftware.net/) with the maximum parsimony algorithm. We identified antimicrobial resistance and virulence genes, multilocus sequence type, staphylococcal cassette chromosome *mec* (SCC*mec*) type, and *spa* type using available pipelines (https://cge.cbs.dtu.dk/services/) and confirmed the virulence and antimicrobial resistance gene profile by DNA microarray. We performed a phylogenetic comparison of S23009-2017 with 22 previously published MRSA ST398 whole-genome sequences and rooted the tree with an outgroup of single-locus Panton-Valentine leukocidin (PVL)–positive variants.

We identified S23009-2017 as a PVL-negative, t011-carrying, MRSA ST398 type V (5C2&5) isolate. *spa* type t011 and SCC*mec* element type V (5C2&5) are frequently identified in LA-MRSA ST398 isolates (*3*,*4*). However, unlike LA-MRSA ST398, which is typically phenotypically multidrug resistant and harbors multiple antimicrobial drug resistance genes, including *tetM* (*5*), S230090-2017 was only resistant to β-lactams (penicillin and oxacillin) and harbored only the *blaZ* and *mecA* antimicrobial drug resistance genes. Furthermore, S23009-2017 harbored the *sak*, *chp*, and *scn* human evasion genes, which are not typically found in LA-MRSA ST398 (*6*).

Phylogenetic analysis showed S23009-2007 had a much closer relationship to the PVL-negative MRSA ST398 isolates from China reported by He et al. than to LA-MRSA ST398 isolates from Australia (Figure) (*7*). These isolates are grouped within the II-GOI (group of interest) clade from the Price et al. study of worldwide MRSA ST398 isolates (*6*).

**Figure:**
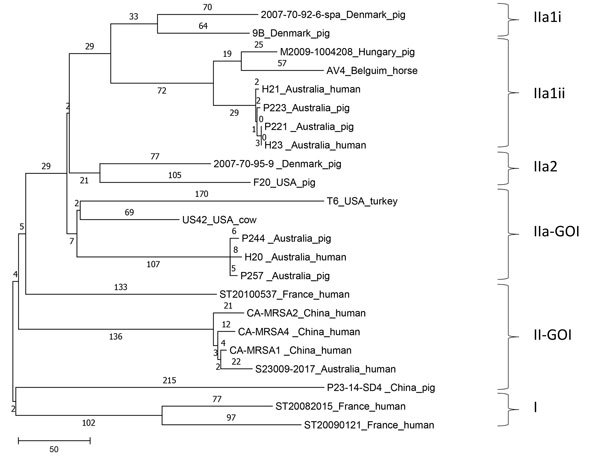
Phylogenetic tree of MRSA sequence type (ST) 398 isolate S23009-2017, recovered from a man in Australia in 2017, and related MRSA ST398 isolates from around the world (*2*,*6*,*7*). The tree was constructed by using single-nucleotide polymorphism differences and is rooted with MRSA ST398 isolates containing a single Panton-Valentine leukocidin locus. Isolates are grouped into clusters as described by Price et al. (*6*). Scale bar represents number of nucleotide substitutions per residue. CA-MRSA, community-associated MRSA; GOI, gene of interest; MRSA, methicillin-resistant *Staphylococcus aureus*.

He et al. reported several cases of severe and fatal infections with community-associated MRSA (CA-MRSA) ST398 and proposed that the strain arose from human-adapted predecessors and not from a livestock-adapted strain. Although all of the China isolates harbored a type V SCC*mec*, only 1 isolate harbored the type V (5C2&5) SCC*mec* element found in S23009-2017. Like S23009-2017, CA-MRSA ST398 isolates from China harbor *sak, chp*, and *scn* genes and lack the *tetM* resistance gene.

MRSA ST398 has not been previously reported to cause serious disease in Australia. Although LA-MRSA ST398 is frequently identified in pig herds in Australia, the isolate from this patient was not LA-MRSA but CA-MRSA and presumably originated in China. Because of immigration irregularities, we were not able to investigate whether the patient had visited China shortly before his illness or if his house companions were from China. Unlike LA-MRSA ST398, CA-MRSA ST398 has been shown to be highly virulent and has become the predominant CA-MRSA circulating in Shanghai, China. Thus, continued monitoring of this strain’s epidemiology and preventing its widespread transmission are essential.
